# Mitochondrial Function and Reactive Oxygen/Nitrogen Species in Skeletal Muscle

**DOI:** 10.3389/fcell.2022.826981

**Published:** 2022-02-21

**Authors:** Ming-Ming Chen, Yan Li, Shou-Long Deng, Yue Zhao, Zheng-Xing Lian, Kun Yu

**Affiliations:** ^1^ College of Animal Science and Technology, China Agricultural University, Beijing, China; ^2^ NHC Key Laboratory of Human Disease Comparative Medicine, Institute of Laboratory Animal Sciences, Chinese Academy of Medical Sciences and Comparative Medicine Center, Peking Union Medical College, Beijing, China

**Keywords:** skeletal muscle, mitochondrial function, mitochondrial dynamics, RONS, oxidative stress

## Abstract

Skeletal muscle fibers contain a large number of mitochondria, which produce ATP through oxidative phosphorylation (OXPHOS) and provide energy for muscle contraction. In this process, mitochondria also produce several types of “reactive species” as side product, such as reactive oxygen species and reactive nitrogen species which have attracted interest. Mitochondria have been proven to have an essential role in the production of skeletal muscle reactive oxygen/nitrogen species (RONS). Traditionally, the elevation in RONS production is related to oxidative stress, leading to impaired skeletal muscle contractility and muscle atrophy. However, recent studies have shown that the optimal RONS level under the action of antioxidants is a critical physiological signal in skeletal muscle. Here, we will review the origin and physiological functions of RONS, mitochondrial structure and function, mitochondrial dynamics, and the coupling between RONS and mitochondrial oxidative stress. The crosstalk mechanism between mitochondrial function and RONS in skeletal muscle and its regulation of muscle stem cell fate and myogenesis will also be discussed. In all, this review aims to describe a comprehensive and systematic network for the interaction between skeletal muscle mitochondrial function and RONS.

## 1 Introduction

Skeletal muscle is a high energy-consuming tissue, the energy requirements during intense contraction increase to 100-fold the consumption of triphosphate (ATP) (Gaitanos et al., 1985). Mitochondria, as the central organelles of skeletal muscle metabolism, provide about 80% of the energy for cell life activities, and the normal function of mitochondria is essential for regulating the metabolic activities of carbohydrates, lipids, and protein homeostasis in organisms. To maintain this high energy demand, the skeletal muscle especially red muscle fibers relies on mitochondrial oxidative phosphorylation (OXPHOS) to produce ATP. In addition to ATP generation, mitochondria also produce several types of “reactive species” as side products, such as reactive oxygen species (ROS) and reactive nitrogen species (RNS), which have attracted interest. Reactive oxygen/nitrogen species (RONS) are part of the normal cellular metabolism at steady-state, and are a group of oxygen/nitrogen-derived molecules and free radicals. The imbalance of RONS and endogenous or exogenous antioxidants causes oxidative stress, and may result in oxidative damage to muscle fibers via apoptosis, autophagy, and inflammation. It was therefore long thought that RONS had adverse health effects. However, the presence of RONS is essential in maintaining muscle functions such as skeletal muscle development, injury repair, muscle mass and mitochondrial biogenesis. Accumulating evidence suggests that RONS is a double-edged sword in living systems (Valko et al., 2007).

From the perspective of skeletal muscle mitochondria, we will review the origin and physiological function of RONS, mitochondrial structure and function, mitochondrial dynamics and the relationship between RONS and mitochondrial oxidative stress. Additionally, the crosstalk mechanism between mitochondrial function and RONS in skeletal muscle and its regulation of muscle stem cell fate and myogenesis will be discussed. In all, this review aims to describe a comprehensive and systematic network for the interaction between skeletal muscle mitochondrial function and RONS.

## 2 Reactive Oxygen/Nitrogen Species and Antioxidants in Skeletal Muscle

### 2.1 Reactive Oxygen and Nitrogen Species

The common forms of intracellular ROS include superoxide anion radical (O_2_^•-^), hydroxyl radical (^•^OH), hydrogen peroxide (H_2_O_2_), and lipid hydroperoxides (LOOH) (Hayyan et al., 2016; Chakrabarty and Chandel 2021). A large variety of ROS are constantly produced in skeletal muscle during resting and contracting (Henríquez-Olguín et al., 2019).

Among them, O_2_^•-^ is the main oxidant molecule produced by adding an electron to molecular oxygen (O2), which is itself a radical. Skeletal muscle has several sources of ROS, multiple organelles, including peroxisomes, endoplasmic reticulum, and mitochondria, are well known to produce O_2_^•-^ (Dickinson and Chang 2011). Many cytosolic enzymes such as NADPH oxidases, and monoamine oxidases also generate O_2_^•-^ locally (Powers and Jackson 2008). As the main source of ATP in the mammalian cells, the mitochondrial electron transport chain (ETC) can produce O_2_^•-^ from at least 11 different sites on both sides of the inner mitochondrial membrane (Shadel and Horvath 2015). Under physiological conditions, the complexes I and III of ECT are currently believed to be the major production sites of O_2_^•-^ (Brand 2010). Due to O_2_^•-^ is too strongly charged to easily cross the inner mitochondrial membrane, it may act a locally role in mitochondria (Valko et al., 2007; Henriquez-Olguin et al., 2020).

**FIGURE 1:**
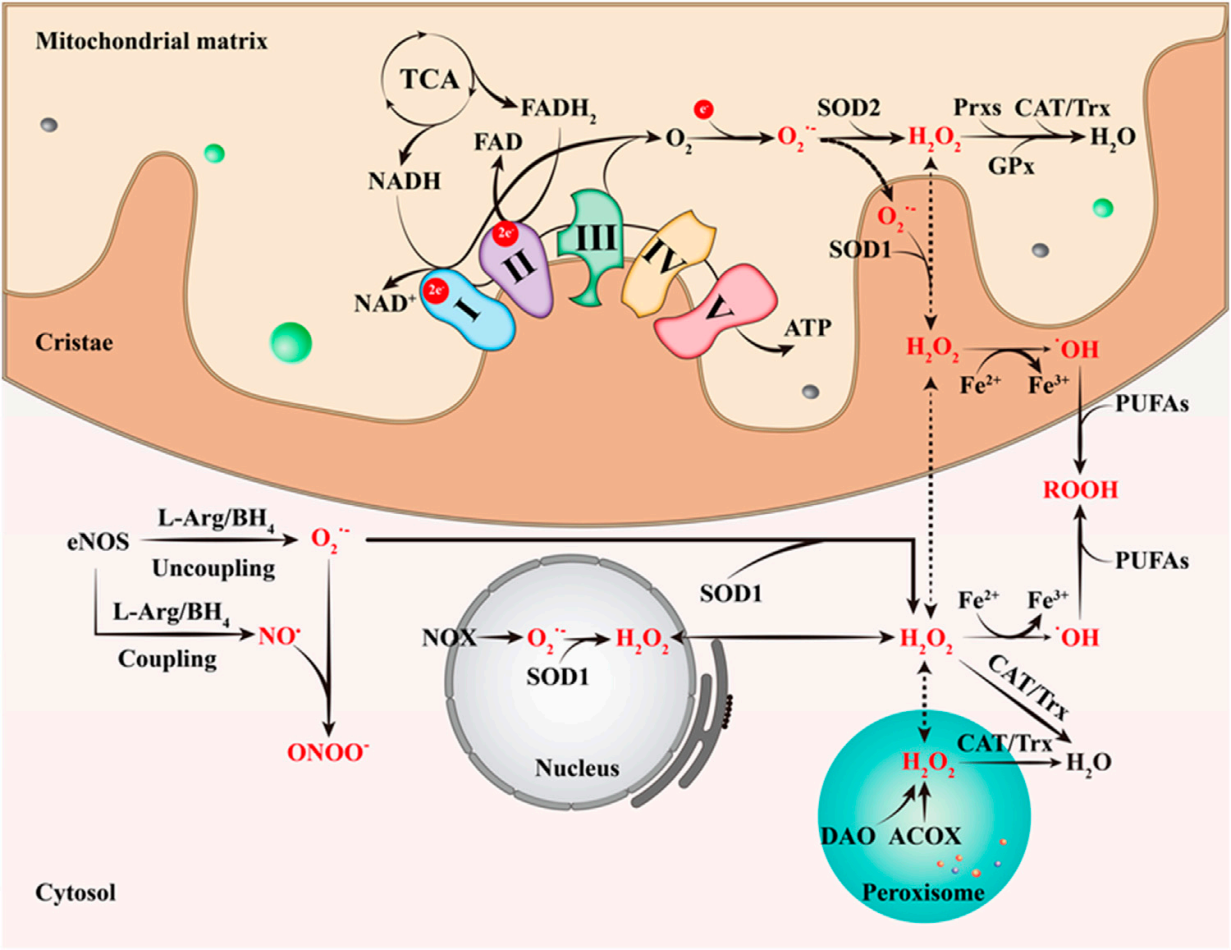
The primary ways of RONS production. The complexes I and III of ECT are currently believed to be the major production sites of O_2_^•-^. O_2_^•-^ is then rapidly and spontaneously converted to H_2_O_2_, which is accelerated by SOD2. Next, Prxs, GPx, CAT and Trx catalyze the conversion of H_2_O_2_ to H_2_O. H_2_O_2_ can easily cross the cell membrane and react with Fe^2+^ to produce ^•^OH, the strongest oxidant in ROS. In addition, SOD1 catalyzes the conversion of the O_2_^•-^ produced by NOX in the nucleus to H_2_O_2_; ACOX and DAO also can generate H_2_O_2_ in peroxisome. This figure is inspired by Chakrabarty, R.P. and N.S. Chandel (Chakrabarty and Chandel 2021). TCA, tricarboxylic acid cycle; SOD1, superoxide dismutase 1; SOD2, superoxide dismutase 2; ATP, triphosphate; Prxs, peroxyredoxin; CAT, catalase; Trx, thioredoxin; GPx, glutathione peroxidase; PUFAs, polyunsaturated fatty acids; ROOH, organic hydroperoxides; DAO, d-amino acid oxidase; ACOX, acyl-CoA oxidase; NOX, NAPDH oxidase; L-Arg, L-arginine; BH_4_, tetrahydrobiopterin.

O_2_^•-^ produced by organelles or cytoplasmic enzymes is rapidly and spontaneously converted to H_2_O_2_, and this process is accelerated by superoxide dismutase (SOD) isoforms (Powers and Jackson 2008) (Figure 1). Because of its relatively lower reactivity, H_2_O_2_ is recognized as the primary redox signaling molecule in the redox regulation of biological activities (Henriquez-Olguin et al., 2020; Veal and Day 2011; Sies 2014). Although the intracellular concentration of H_2_O_2_ is maintained in the low level range (1–100 nM), the overall cellular concentration is much higher than that of O_2_^•-^, at 10^–3^ nM (Sies and Jones 2020). At the same time, its concentration in normal cells is in a strict dynamic equilibrium state. Metabolic activity or various stressors, such as chemokines, growth factors, and physical stressors, stimulate the generation of H_2_O_2_, while its removal is driven by an effective reduction system (Sies and Jones 2020; Parvez et al., 2018); that is, peroxyredoxin (Prxs) and glutathione peroxidases (GPx) catalyze the conversion of H_2_O_2_ to H_2_O (Rhee and Kil 2017; Brigelius-Flohé and Flohé 2020) (Figure 1). As a signaling molecule, H_2_O_2_ directly oxidizes specific sulfur-containing amino acids (cysteine and methionine), which are crucial for protein function, activity, stability, subcellular localization, and interactions, thus regulating various physiological processes in cells and organs, such as cell activation, proliferation, differentiation, migration, fusion and angiogenesis (Tan and Suda 2018; Zhang et al., 2019). It is worth noting that mitochondrial nicotinamide nucleotide transhydrogenases (NNT) are also involved in clearing cellular H_2_O_2_, which is achieved by shifting the reducing equivalents from NADH to NADPH (Hanschmann et al., 2013; Mailloux 2018). H_2_O_2_ can easily cross the cell membrane and react with Fe^2+^ to produce ^•^OH, which is the neutral form of hydroxyl ion (OH^-^) (Henriquez-Olguin et al., 2020) (Figure 1). Although its half-life (10^–9^ s) *in vivo* is very short, ^•^OH is the strongest oxidant in ROS due to its high reactivity and the lack of specific scavenging enzymes (Pastor et al., 2000).

RNS, including nitric oxide (NO), peroxynitrite, and nitroxylanion, is also an important class of oxidative bio-signal molecules. Nitric oxide synthases (NOS) are a group of enzymes that produce NO from L-arginine (L-Arg), O_2_ and NADPH. Skeletal muscle contains three NOS subtypes, which are neuronal NOS (nNOS), inducible NOS (iNOS) and endothelial NOS (eNOS) (Hussain et al., 1985; Ohkoshi et al., 1997; Tengan et al., 2012). NOS enzymes are also regards as a origin of O_2_^•-^ production that occurs when NOS is uncoupled from its substrate L-Arg and its cofactor BH_4_ (Luo et al., 2014; Steinz et al., 2020). NO^•^ is a radical because it contains one unpaired electron on the antibonding (Valko et al., 2007). It is known that NO^•^ is produced by eNOS, which metabolize arginine to citrulline with the formation of NO^•^ through a five electron oxidative reaction (Ghafourifar and Cadenas 2005). NO^•^ plays an important role in many physiological processes, including blood pressure regulation, smooth muscle relaxation and immune regulation (Valko et al., 2007). During an inflammatory reaction, however, immune cells produce O_2_^•-^ and NO^•^, reacting together to generate a large amount of a highly reactive and strong oxidant, peroxynitrite anion (ONOO^-^), and leading to DNA fragmentation and lipid oxidation (Carr et al., 2000).

Although mitochondrial RONS are involved in numerous physiological processes (Görlach et al., 2015; Latorre-Pellicer et al., 2016; Mills et al., 2016; Acín-Pérez et al., 2020), and also link the tissue homeostasis with disease progression (Chouchani et al., 2016; Harris and DeNicola 2020). However, the production mechanisms of mitochondrial RONS has not been fully revealed (Murphy 2009; Dröse and Brandt 2012; Wong et al., 2017). As mentioned above, complex I and complex III are the main sources of mitochondrial and intracellular RONS. In general, the potential sites of RONS production are triggered depending on the respiration substrates, membrane potential (∆*Ψ*_m_) and, if exist, the inhibitor. Under normal conditions, the coupled respiration on glutamate/malate or pyruvate/malate will activate the tricarboxylic acid (TCA) cycle enzymes 2-oxoglutarate dehydrogenase (OGDH), malate dehydrogenase (MDH), and pyruvate dehydrogenase (PDH), and maintains a low ∆*Ψ*_m_ as complex V is generating ATP. OGDH, PDH, and MDH reduce NAD^+^ to NADH, which is a substrate of complex I (Hernansanz-Agustin and Enriquez 2021). As the electrons flow down the ETC, they eventually reach complex III and IV. In this case, the production of RONS is low but measurable (Murphy 2009; Wong et al., 2017). Succinate is the substrate of the TCA cycle enzyme succinate dehydrogenase (SDH), also known as complex II. As a representative of a variety of FAD dependent enzymes in the inner mitochondrial membrane, SDH can reduce ubiquinone (Scialò et al., 2017). When reducing potential is provided by complex II or other FAD-dependent enzymes, mitochondria over reduce ubiquinone (Robb et al., 2018). In addition, the electrons can reflux through complex I, reducing NAD^+^ into NADH and producing superoxide under conditions of mitochondrial hyperpolarization (Robb et al., 2018). This process is known as reverse electron transfer. The reverse electron transfer is the mode that produces the highest level of RONS and has been observed in both physiological and pathophysiological conditions (Chouchani et al., 2014; Guarás et al., 2016; Mills et al., 2016; Robb et al., 2018). Independent of the electron source, complex III can generate RONS after being inhibited by specific molecules (Quinlan et al., 2013). In addition, Ca^2+^ is the most famous second messenger, the interaction between elevated intracellular calcium levels and inflammation is considered to be one of the causes of significant oxidative stress (Wolf et al., 2017). The increase of cytosolic calcium level leads to the increase of mitochondrial calcium concentration, which would affect the synthesis of ATP (Brookes et al., 2004; Feissner et al., 2009). During increased ATP production, higher oxygen consumption and enhanced electron flow through the electron transport chain increased the content of ROS in muscles (Brookes et al., 2004; Feissner et al., 2009). A recent study demonstrated that Na^+^ acts as a second messenger to regulate OXPHOS function and the production of ROS by modulating the fluidity of the inner mitochondrial membrane (Hernansanz-Agustín et al., 2020).

### 2.2 Antioxidants

Although RONS is continuously produced in cells, especially during skeletal muscle contraction and physical exercise, they have an adaptive defense system to control the level of RONS, which is essential to balance RONS content in muscle (Jiang et al., 2020). Any substance that scavenges oxygen free radicals or inhibits the oxidation process in cells is considered as antioxidant (Diaz de Barboza et al., 2017). Depending on their source, antioxidants can be divided into endogenous and exogenous. The endogenous antioxidant defense system consists of the enzymatic antioxidants and non-enzymatic antioxidants. The enzymatic antioxidants include superoxide dismutase (SOD), glutathione peroxidase (GPx), catalase (CAT), and thioredoxin (Trx), while the non-enzymatic antioxidants are represented by *α*-tocopherol (Vitamin E), glutathione (GSH) and bilirubin, and other antioxidants (Valko et al., 2007; Oyewole and Birch-Machin 2015). Exogenous antioxidant defense system contains ascorbic acid (Vitamin C), carotenoids, and flavonoids (Oyewole and Birch-Machin 2015).

Endogenous and exogenous antioxidants are widely distributed in the body. However, their distribution in cells is different, most of them are located in cytosol and a few in mitochondria. In mitochondria, the antioxidant defense system also articulates on various levels. Most superoxide or hydrogen peroxide production sites release their product to the mitochondrial matrix. In the matrix, the enzyme that converts the O_2_^•−^ to H_2_O_2_ is SOD2, which is a tetramer containing one manganese atom per subunit (Fridovich 1995). A recent study suggested that SOD2 broadcasts the redox signals generated by mitochondria to distant sites in the cytosol, nucleus or even outside the cell (Palma et al., 2020). In the mitochondrial intermembrane space, SOD1, which dismutated the released superoxide (Okado-Matsumoto and Fridovich 2001). Due to its active center includes a Cu ion and a Zn ion, SOD1 also named CuZnSOD. The SODs-catalyzed reaction of O_2_^•−^ dismutation is in competition with the reaction between O_2_^•−^ and NO^•^, which prevents O_2_^•−^ from reacting with NO^•^ to form the highly reactive peroxynitrite (Radi, 2018). After O_2_^•−^ is converted to H_2_O_2_, it is removaled by CAT, Trx and GPx systems. The CAT is found mainly in peroxisomes, it breaks down two hydrogen peroxide molecules into one molecule of O_2_ and two molecules of H_2_O in a two-step reaction (von Ossowski et al., 1993). The GPx and Prx metabolize most of the H_2_O_2_. Their activities depend on the thiol groups of the residues of cysteine of reduced GSH and Trx, respectively. Up to now, eight isoforms of GPxs have been identified. The mammalian GPx1, GPx2, GPx3, and GPx4 are seleno-proteins containing selenocysteine in the catalytic center, while GPx6 is a seleno-protein only in humans (Napolitano et al., 2021). GPx5 contains cysteine instead of selenocysteine in the active center, while GPx7 and GPx8 are cysteine-GPxs with low GPx activity (Brigelius-Flohé and Flohé, 2017). Among those GPx, only GPx1 and GPx4 exist in mitochondria. GPx1, which exists in the cytosol and mitochondria, is a widely expressed homologous tetramer. It works in a similar way to CAT by breaking down H_2_O_2_, but slowly and with a high affinity. The GSH is the main non-enzymatic regulator of intracellular redox homeostasis. GPx1 can be converted to oxidized glutathione (GSSG) using GSH as a reducing agent (Brigelius-Flohé and Maiorino, 2013). GPx4 is a monomer that can reduce hydroperoxides in complex lipids (Brigelius-Flohé and Maiorino, 2013). GSSG produced by GPx activity cannot leave the mitochondria, it is recycled back to GSH through glutathione reductase (GR), which uses reduced NADPH as a hydrogen donor (Couto et al., 2016). The thioredoxin system includes thioredoxin reductase (TrxR), Trx, and Prxs. TrxR transfers electrons from NADPH to Trx, and the reduced Trx donates an electron to Prxs, thereby reducing H_2_O_2_ to H_2_O (Holmgren and Lu, 2010). The Trxs contain a thiol motif of the preserved active site. Among them, Trx1 is found mainly in the cytoplasm and nucleus, while Trx2 is exist in the mitochondria (Ribas et al., 2014). With comprise six isoforms in the mammalian cell, Prxs are a large family of thiol-dependent peroxidases (Rhee and Kil, 2017). Among them, Prx1, 2 and 6 are exist in the cytoplasm, Prx4 in the endoplasmic reticulum, Prx3 in mitochondria and Prx5 in various compartments, including peroxisomes and mitochondria (Wood et al., 2003). Vitamin C, also named ascorbic acid, is a water-soluble vitamin. It has been proved that its concentration in mammalian mitochondria is increased in dietary Vitamin C supplementation (Li et al., 2001; Ramanathan et al., 2003). This increase depends on the existence of special mitochondrial uptake mechanisms. The carrier of the oxidized vitamin, dehydroascorbic acid (DHA), was initially identified as the facilitative glucose transporter 1 (GLUT1) (Kc et al., 2005). The DHA that enters into the mitochondria is reduced and accumulated as mitochondrial ascorbic acid, which can inactivate RONS, thus protecting mitochondrial genome and membranes from oxidative damage (Kc et al., 2005). Vitamin E is the main lipid-soluble antioxidant in cells. It consists of tocopherols and tocotrienols that contain a chromanol ring with a 13-carbon chain at the C2 position. Vitamin E plays an antioxidant role in different ways. It can inactivate oxygen singlet by quenching, and one molecule of *a*-tocopherol can deactivate up to 120 oxygen singlet before its degradation (Mukai et al., 2018). In addition, Vitamin E is a powerful, chain-breaking antioxidant that can chemically scavenge oxygen singlet and lipid peroxyl radicals. This protective effects of Vitamin E from oxidative damage also depends on its ability to scavenge superoxide radicals, thereby down-regulating the generation of mitochondrial ROS (Gotoh and Niki 1992).

In general, the oxidative stress occurs rarely in cells, and the endogenous antioxidant system in the cells will clear RONS. However, the antioxidant capacity of the antioxidant system is limited, once severe oxidative stress occurs, the antioxidant system will not be completely to clear excess RONS, which will result in the accumulation of RONS and cause oxidative damage to intracellular lipids, DNA, and proteins (Jiang et al., 2020).

## 3 Mitochondrial Structure and Function in Skeletal Muscle

Skeletal muscle fibers contain a large number of mitochondria, which function in ATP synthesis through oxidative phosphorylation to provide energy for muscle contraction. Mitochondrial dysfunction is closely associated with muscle atrophy, metabolic syndromes and other muscle diseases. A large amount of evidence has indicated that maintaining the normal function of mitochondria is essential for the development of skeletal muscle (Calvani et al., 2013; Bhatti et al., 2017). Generally, mitochondria are enclosed and wrapped by inner and outer membranes. The two membranes separate the internal space of mitochondria from the cytoplasm and divide the internal space into two membranous spaces, thus forming the basic scaffold of mitochondrial structure. The inner membrane contains the inner boundary membrane and cristae membrane, which are connected via tubular cristae junctions. The cristae are dynamic invaginations and infoldings of the inner membrane, which harbors the respiratory chain complexes composed of the ETC (Pileggi et al., 2021). A variety of transport proteins are embedded in the outer membrane, and forms the larger water-phase voltage-dependent anion channel (VDAC) across the lipid bilayer, leading to the appearance of multiple pores with a diameter of 2–3 nm in the outer membrane. Thus, the ATP, NAD, Coenzyme A (CoA) and other small molecules (<10 kDa) can pass freely through the outer membrane, rendering the intermembrane space chemically similar to the cytoplasm (Nicholls 2009; Hood et al., 2019). Conversely, the permeability of the inner membrane is very small, which is necessary for the establishment of proton electrochemical gradient and ATP synthesis. In addition, the inner membrane is the key site of mitochondrial oxidative phosphorylation because it contains the oxidative phosphorylation protein complexes of ETC (Hood et al., 2019). Specifically, the enzyme complexes I-IV on the inner membrane catalyzes electron transfer through a series of redox reactions to produce electrochemical gradient or protonmotive power, which is used to drives F_1_F_0_-ATP synthase to form ATP as protons return to the matrix (Yadava and Nicholls 2007; Pileggi et al., 2021). The TCA cycle and *β*-oxidation in the matrix can catabolize metabolites from cytoplasm, fatty acids and other high-energy biomolecules to generate reducing equivalents/electrons that drive the ETC (Pileggi et al., 2021).

Now known, the morphology of mitochondria varies greatly among different tissues, depending on its highly dynamic fusion and fission process. In skeletal muscle, mitochondria exist as an interconnected network, commonly known as the reticular structure, depending on their position in muscle fibers (Ogata and Yamasaki 1997; Hood et al., 2019). Mitochondrial networks are connected in the form of the proton-motive force, which enables rapid communication and distribution of potential energy throughout the cell (Glancy et al., 2015). The mitochondrial networks of cardiac and skeletal muscle is divided into subnetworks composed of numerous mitochondria, which are connected by rich contact sites at highly specific inter mitochondrial junctions (Glancy et al., 2017). The regional mitochondrial subnetworks limit the effects of local dysfunction on cells, while the dynamic disconnection of damaged mitochondria allows the remaining mitochondria to resume normal function within seconds (Glancy et al., 2015; Glancy et al., 2017). In general, mitochondria in skeletal muscle are subdivided into two different subpopulations with different morphological and biochemical characteristics (Picard et al., 1985). The mitochondria that are below the sarcolemma membrane, proximal to the capillary and nuclei are important in providing ATP for membrane active transport and gene transcription (Ferreira et al., 2010). Those located between the myofibrils near the Z-line of sarcomere play a critical role in providing ATP to contractile filaments to accelerate contraction (Hood et al., 2019). The second type of mitochondria can directly come into contact with the transverse tubules, and especially the Ca^2+^ releasing units of sarcoplasmic reticulum (Boncompagni et al., 2009). The ER-mitochondrial interface is central to calcium signaling, organellar dynamics, it hosts a nanodomain of H_2_O_2_, which is induced by cytoplasmic Ca^2+^ spikes and exert a positive feedback on calcium oscillations (Booth et al., 2016). A recent study showed that individual mitochondria initiate local retrograde signaling through miniature oxidative bursts and, upon metabolic or apoptotic stress, may also amplify signals to the rest of the cell (Booth et al., 2021). Thus, ER-mitochondrial H_2_O_2_ nanodomains represent a novel component of inter-organelle communication, they may be involved in the regulation of intracellular Ca^2+^ signaling, maintain Ca^2+^ homeostasis and regulate skeletal muscle contraction and mitochondrial activities such as the production of mitochondrial RONS (Booth et al., 2016; De La Fuente et al., 2016; Booth et al., 2021). Interestingly, proton leak can cause oxygen consumption through a mechanism independent of ATP synthase. This process contributes to about 30% of the oxygen consumption of cells, and occurs through the basal proton leak, uncoupling proteins (UCPs), and mitochondrial ADP/ATP carrier (AAC) (Brand et al., 1994; Bernardi 2019; Bertholet et al., 2019). The mitochondrial uncoupling reduces mitochondrial ∆*Ψ*_m_, maintains electron flow through ETC and minimizes electronic “escape” to against RONS (Brand 2000; Pileggi et al., 2021). In addition, numerous studies have revealed other functions of mitochondria beyond the aforementioned. They contribute to regulating nuclear gene expression (Chae et al., 2013), synthesizing essential macromolecules including heme molecules (Lill et al., 2012), determining the fate of muscle stem cells (Bhattacharya and Scimè 2020), and releasing cell pro-apoptotic factors and immunogenic pro-inflammatory molecules (Zhang et al., 2010; Lill et al., 2012).

## 4 Mitochondrial Dynamics in Skeletal Muscle

Mitochondria are highly dynamic organelles that are continuously undergoing ultrastructural remodeling, forming a tubular network in myoblasts under normal conditions. The number, size, morphology, and localization of the mitochondrial reticulum, which regulates mitochondrial activity and abundance, relies on the dynamic interaction among mitochondrial fusion, fission, cristae remodeling, and movement events (Mishra and Chan 2016; Eisner et al., 2018; Pileggi et al., 2021). The coordinated action among these events is often known as mitochondrial dynamics, which is an important characteristic of myogenesis and skeletal muscle regeneration. In addition, mitochondrial content can be regulated by mitochondrial biogenesis (*de novo* synthesis) and mitophagy removing damaged mitochondria. Mitochondrial fusion and fission serve as a bridge between biogenesis and mitophagy. Mitochondrial fusion prevents mitophagy, while fission is a key step preceding mitophagy (Rahman and Quadrilatero 2021). In short, mitochondrial biogenesis, kinetics and mitophagy regulate the quantity, quality, and morphology of mitochondria, which are the main quality control mechanisms of mitochondria.

### 4.1 Fusion

**FIGURE 2:**
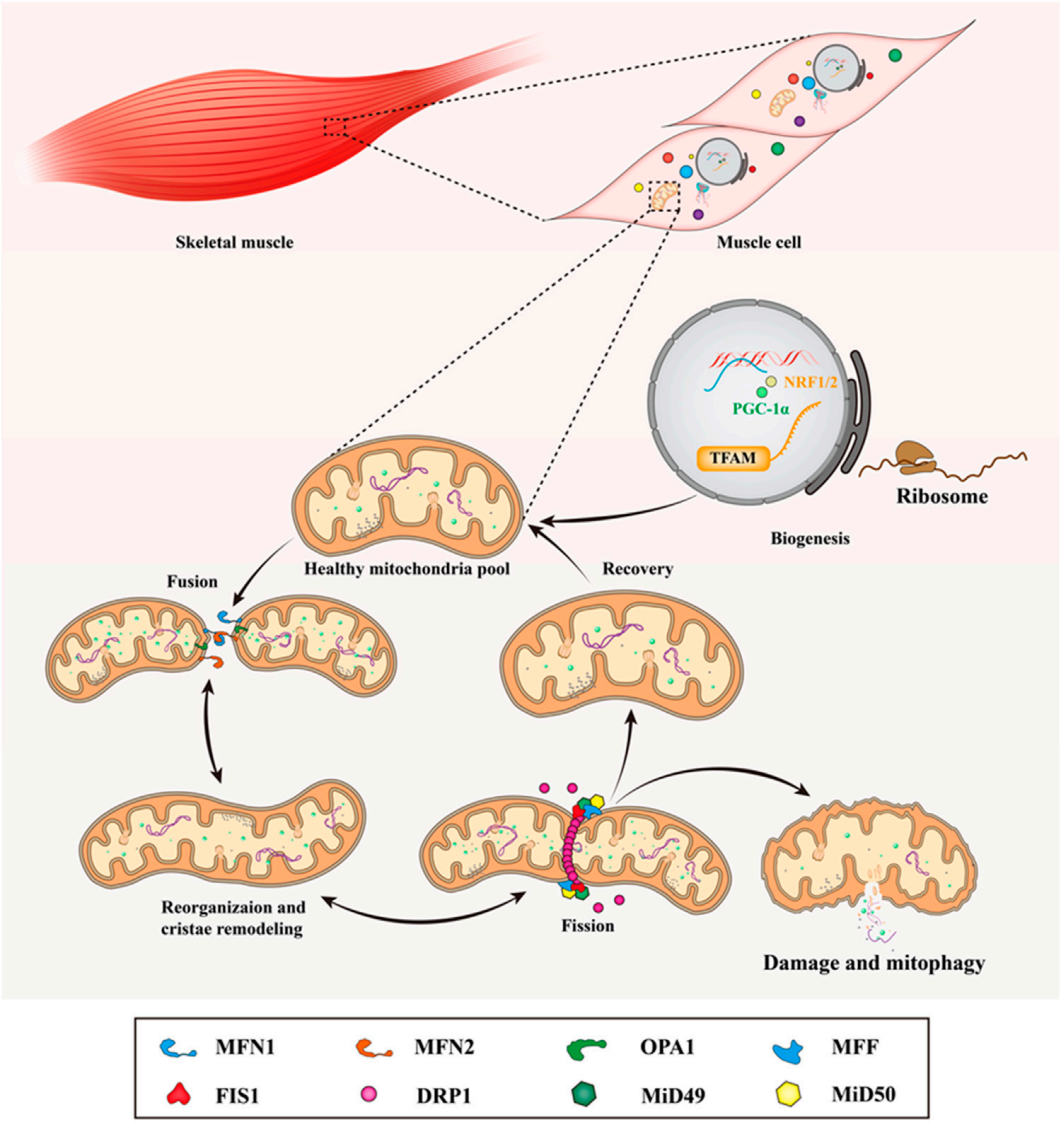
Mitochondrial dynamics in skeletal muscle. In mammalian skeletal muscle, the life cycle of the mitochondria under through biogenesis, fusion, reorganization and cristae remodeling, fission, damage and mitophagy. When mitochondria undergo fusion, MFN1/2 mediates the fusion of the outer mitochondrial membrane, while the fusion of the inner mitochondrial membrane is initiated by OPA1. At the division sites of the outer mitochondrial membrane, DRP1 is recruited and assembled to form a ring-like spiral structure by the MFF, FIS1, MiD49, and MiD51, then it works in conjunction with MFF and FIS1 to wrap around and constrict the mitochondria to promote the fission of mitochondrial network. This figure is inspired by Hood, D.A., et al. (Hood et al., 2019).

Mitochondrial fusion is the merging of two or more mitochondria in close contact to form one mitochondrion, which leads to mitochondrial enlargement with mixed compartments. In mammalian skeletal muscle, the mitochondrial fusion mechanism is driven by three main GTPases belonging to the dynamin superfamily (Tilokani et al., 2018) (Figure 2). Among them, the fusion of the outer mitochondrial membrane is mediated by mitofusin 1/2 (MFN1/2), while the fusion of the inner mitochondrial membrane is initiated by optic atrophy 1 (OPA1) (Eisner et al., 2018) (Figure 2). GTP hydrolysis induces the conformational change of the MFN1/2 oligomer, which accumulates and anchors on the adjacent mitochondrial outer membrane, then pulling and fusing the mitochondrial outer membrane (Tondera et al., 2005; Hoppins et al., 2011). After the outer membrane fusion, the long isoform of OPA1 (L-OPA1) mediates fusion of the inner mitochondrial membrane through the interaction between heteropolymer and cardiolipin, which also depends on the hydrolysis of GTP (Cho et al., 2017).

Usually, mitochondrial fusion is controlled by post-transcriptional modifications. For instance, peroxisome proliferator-activated receptor gamma coactivator 1-alpha (PGC-1α) and estrogen receptor (ER) can regulate the transcription of MFN1/2 by binding to their promoter region (Martin et al., 2014). In addition, high levels of oxidized glutathione produce disulfide bonds and induce the dimerization of MFN1/2, and form the redox signal during this process to stimulate mitochondrial fusion (Shutt et al., 2012). During aging, the expression of MFN2 decreases and abnormal mitochondria accumulated during aging, which results in sarcopenia. MFN2-deficient muscles are characterized by mitochondria dysfunction, increased RONS production and ER stress (De Mario et al., 2021). A muscle-specific MFN1 and MFN2 double gene knockout mice models confirmed that the mutant mice showed severe mitochondrial DNA deletion and mitochondrial dysfunction in muscle, which strongly increased the lethality of the mutant mice (Chen et al., 2010), suggesting that MFN1/2 plays a dual role in protecting the integrity of mtDNA and maintaining its function by ensuring the remixing of mtDNA among mitochondria. Skeletal muscle specific deletion of OPA1 during embryonic development caused the fusion disorder of the inner mitochondrial membrane and neonatal death within 9 days after birth (Tezze et al., 2017). Newborn mice were smaller than the control group, and their muscle size decreased from the reduction in fiber size (Tezze et al., 2017). Of note, the muscle atrophy can still be observed in a mild decline model of OPA1 protein. However, the non-negligible residual OPA1 expression will produce a beneficial metabolic effect, which is the result of ER stress and skeletal muscle secretion of FGF21 to increase metabolic rate and improve insulin sensitivity (Pereira et al., 2017). All these evidences emphasize the critical function of MFN1/2 and OPA1 in skeletal muscle mitochondrial fusion.

### 4.2 Fission

Mitochondrial fission, is a process that involves the mitochondrial membrane constriction, and scission, resulting in two mitochondria. It is initiated to remove damaged and dysfunctional mitochondria from the mitochondrial pool during the loss of ∆*Ψ*_m_ and oxidative stress caused by RONS (Chen et al., 2010; Twig et al., 2008), although a detailed molecular mechanism of mitochondrial fission remains the Riddle of the Sphinx. However, it is generally acknowledged that mitochondrial fission is regulated by GTPase dynamin-related protein 1 (DRP1) (Fonseca et al., 2019). At the division sites of the outer mitochondrial membrane in mammals, DRP1 is recruited and assembled to form a ring-like spiral structure by the adaptor proteins mitochondrial fission factor (MFF), mitochondrial fission protein 1 (FIS1), and mitochondrial dynamics proteins of 49 and 51 kDa (MiD49 and MiD51). It then works in conjunction with MFF and FIS1 to wrap around and constrict the mitochondria to promote the fission of the mitochondrial network (Ingerman et al., 2005; Otera et al., 2010; Losón et al., 2013) (Figure 2). Among them, MFF is the main adaptor protein, which can stimulate DRP1 GTPase activity during mitochondrial and peroxisomal fission (Gandre-Babbe and van der Bliek 2008). The recruitment of DRP1 to the outer membrane by FIS1 may be cell-type-specific or cell signaling cues-specific, and MiD49 and MiD51 can independently recruit DRP1 and provide scission specificity (Losón et al., 2013; Palmer et al., 2013). Downregulation of MiD49 and MiD51 promotes mitochondrial elongation, while their overexpression causes a fragmented network (Osellame et al., 2016). Aside from the aforementioned proteins, recent studies have also suggested that cellular components such as namely ER and the cytoskeleton are involved in mitochondrial division. Specifically, the precise locations of DRP1 recruitment and mitochondrial contraction point depend on the ER and contact sites (Friedman et al., 2011; Korobova et al., 2013; Li et al., 2015). Of note, only DRP1 is required for full mitochondrial scission, but not dynamins (Fonseca et al., 2019). Similar to the outer mitochondrial membrane, the contraction mechanism of the inner mitochondrial membrane is also not completely revealed. A study suggested that the overexpression of inner mitochondrial membrane protein mitochondrial fission process 1 (MTFP1) can accelerate mitochondrial fission whereas the deletion of MTFP1 causes excessive fusion (Fröhlich et al., 2013). In addition, the tethering effect of inner membrane mitochondrial protein (IMMT) is neutralized by Ca^2+^ flowing into the mitochondrial matrix, which results in a separation of the inner and outer membrane (Casuso and Huertas 2020; Zhang et al., 2021). And, the untethering of the mitochondrial membranes may also facilitate the fission of inner mitochondrial membrane (Casuso and Huertas 2020).

Muscle atrophy implies mitochondrial fragmentation and organelle removal by the autophagy-lysosome system, and mitochondrial fragmentation itself is sufficient to trigger muscle atrophy in an AMPK-dependent manner (Romanello et al., 2010; De Mario et al., 2021). The muscle mass decreases in mice with skeletal muscle-specific DRP1 overexpression, whereas skeletal muscle-specific DRP1 knockout triggers changes in mitochondrial shape, structure and function, leading to severe muscle atrophy (Favaro et al., 2019; Dulac et al., 2020). Furthermore, systemic DRP1 knockout animals died rapidly within the first month of age (Ishihara et al., 2009). Interestingly, the DRP1^−/−^ in skeletal muscle causes the imbalance of intracellular Ca^2+^ homeostasis, which is the most evident feature before muscle wasting (Favaro et al., 2019). The restoration of physiological mitochondrial Ca^2+^ uptake reduces muscle atrophy caused by DRP1 deletion (Favaro et al., 2019). Phosphorylation and dephosphorylation of DRP1 serine (Ser) residues can regulate mitochondrial fission. For example, the phosphorylated Ser^616^ residue contributes to mitochondrial fragmentation in cardiac myoblasts (Xu et al., 2016). Alternatively, mitochondrial depolarization dephosphorylates Ser^673^ residue in a Ca^2+^-dependent manner, resulting in DRP1 activation and mitochondrial disruption (Cereghetti et al., 2008). In summary, DRP1 seems to be a gatekeeper of mitochondrial fission, and its key molecular regulation mechanism is to mark a dysfunctional mitochondrial section and the receptors that recruit DRP1 to mitochondria.

### 4.3 Mitochondrial Cristae Remodeling

The cristae shape is a key regulator of the assembly of mitochondrial respiratory chain super-complexes (RCSs), and mitochondrial OXPHOS activity is highly dependent on cristae shape ETC organization (Cogliati et al., 2013; Cogliati et al., 2016). Skeletal muscle mitochondrial cristae density is responsible for increasing mitochondrial respiration and cell function, and may help to improve systemic oxygen consumption (Nielsen et al., 2017). In addition, it is generally accepted that RCSs are assembled in response to cellular energy drops, and that RCSs assembly contributes to maintaining the production of RONS at a high physiological level (Maranzana et al., 2013; Guarás et al., 2016). These results indicate that RCSs can better mitochondrial respiration and prevent excessive RONS production.

For a long time, cristae have been considered to be mainly a static entity under specific physiological conditions (Kondadi et al., 2020a). Recently, advanced super-resolution nano-microscopy technology has revealed that the cristae are an independent bioenergy unit, which is highly dynamic and remodel on a timescale of seconds (Wang et al., 2019; Wolf et al., 2019; Kondadi et al., 2020b). The cristae remodeling regulates mitochondrial network morphology and mitochondrial function, which is essential for effective respiration, apoptosis, and quality control in cells. Several protein complexes such as the mitochondrial contact site and cristae organizing system (MICOS), F_1_F_0_-ATP synthase and OPA1, interact with inner membrane phospholipids to organize the ultrastructure of the inner membrane, promote the formation, maintenance, and stability of cristae membranes in response to cell requirements (Mukherjee et al., 2021). Mutations or deletions of specific proteins or complexes lead to abnormal defects in cristae structure and impaired mitochondrial function, which may result in altered of RONS levels or Ca^2+^ signaling (Gottschalk et al., 2018; Gottschalk et al., 2019). Aside from regulating the fusion of the inner mitochondrial membrane, OPA1 is also a regulator of cristae morphology and a key factor in maintaining cristae structure. When the cristae are condensed, OPA1 maintains the independent remodeling of cristae fusion, and promotes effective ETC electron transfer through the assembly of RCSs. There is a complicated relationship between OPA1 levels, cristae morphology, and apoptosis. The absence of OPA1 causes the accumulation of swollen cristae and expansion of crista junctions (Frezza et al., 2006). The cell apoptosis induces a widening of cristae, accompanied by unbalanced oligomerization of OPA1, resulting in an increased released of cytochrome c (Cyt c) (Frezza et al., 2006). Acute ablation of OPA1 leads to an increase in the cristae width, accompanied by the loss of RCSs assembly, resulting in respiratory chain inefficiency (Cogliati et al., 2013). Reciprocally, the overexpression of OPA1 restores cristae width and RCSs assembly (Cogliati et al., 2016). These results indicating that OPA1-mediated cristae remodeling regulates energy conversion through OXPHOS. In addition, MICOS stabilizes cristae junctions by providing membrane curvature and establishing contact sites with other membrane protein complexes (Mukherjee et al., 2021). Furthermore, the MICOS complex can directly or indirectly interact with other proteins and play a critical role in maintaining mitochondrial ultrastructure. For example, DNAJC11 helps to maintain proper cristae morphology by interacting with the peripheral MICOS complex (Violitzi et al., 2019).

In short, cristae remodeling can play a crucial role in mitochondrial quality control. Although we are beginning to understand the emerging role of different molecular in cristae dynamics, it is undeniable that the research in the cristae dynamics field is just emerging.

## 5 Coupling Mechanisms Between Reactive Oxygen/Nitrogen Species and Mitochondrial Dynamics

Fragmentation of the mitochondrial network leads to mitochondrial dysfunction, which is manifested by loss of ∆*Ψ*_m_, metabolic shift to glycolysis, decreased respiration and OXPHOS, and increased mitochondrial RONS formation (Nagdas and Kashatus 2017). Higher oxidant levels can open mitochondrial permeability transition pores, further stimulating oxidant generation, which is called “ROS-induced ROS release” and generally linked to apoptosis (Zorov et al., 2014). Here, we focus on the coupling between mitochondrial dynamics and RONS. Parallel changes in RONS levels and mitochondrial dynamics have been reported in many experimental studies. For example, primary fibroblasts cells with greatly reduced Cl activity show a fragmented mitochondrial phenotype and greatly increased RONS levels, while cells with a moderately reduced Cl activity indicate normal mitochondrial morphology and moderately increased RONS levels (Blanchet et al., 2011). With mitochondrial fragmentation, MFN1/2, not OPA1 or FIS1, can be ubiquitinated by exogenous H_2_O_2_ in fibroblasts (Rakovic et al., 2011). Conversely, the antioxidant Trolox can reduce ROS levels in fibroblasts, trigger MFN2-dependent mitochondrial filamentation and increase OXPHOS protein expression and enzymatic activity (Distelmaier et al., 2012). ∆*Ψ*_m_ is an important factor determining mitochondrial structure. Within the physiological values of ∆*Ψ*_m_, mitochondria are interconnected and elongated. However, at both low and high extremes of ∆*Ψ*_m_, which are respectively caused by mitochondrial dysfunction and metabolic stimulation such as high glucose, the mitochondria network displays a fragmented phenotype accompanied by ETC to generate RONS (Brillo et al., 2021). Exposing C2C12 myocytes to exogenous H_2_O_2_ can trigger ∆*Ψ*_m_ depolarization and stimulate mitochondrial fragmentation, involving an increase of DRP1 activity, suggesting that RONS-induced ∆*Ψ*_m_ depolarization may be the cause of mitochondrial fragmentation (Fan et al., 2010; Iqbal and Hood 2014). In addition, muscle factors secreted by skeletal muscle can control mitochondrial dynamics and are actively involved in systemic energy homeostasis, so it may also act as a regulator of RONS production in cells (Pang et al., 2021). For example, MSTN has been proved as a pro-oxidant and signals to generate ROS in skeletal muscle, it stimulates mitochondrial division by regulating the expression of DRP1 and FIS1 (Manfredi et al., 2019; Aravena et al., 2020). In fact, the regulation of myokines in RONS production and mitochondrial dynamics has been reported in many cell types. Here, only myokines involved in skeletal muscle are listed in Table 1. The above results indicate that mitochondrial ultrastructure is closely related to the production of RONS. In other words, there is a complex coupling relationship between cellular redox homeostasis and the regulation of mitochondrial quality control. On the one hand, high levels of RONS, if not offset by an efficient antioxidant system, it will induce a stress response and activate the mitochondrial fission mechanism in many tissues including skeletal muscle, and promote mitochondrial fragmentation, swelling or shortening, whereas the diminished RONS contributes to mitochondrial filamentation. On the other hand, the abnormal mechanism of mitochondrial dynamics observed in the consumption of MFN1/2 leads to enhanced generation of RONS, which may worsen mitochondrial health and further aggravate oxidative stress, forming a self-sustaining vicious cycle (Muñoz et al., 2013; Jezek et al., 2018).

**TABLE 1:** The regulation of myokines in RONS and mitochondrial dynamics in skeletal muscle.

Myokine	Mitochondrial dynamics	RONS
FGF21	Promotes mitochondrial fragmentation Oost et al. (2019)	Inhibits RONS production Pang et al. (2021)
IL-6	Promotes mitochondrial biogenesis Wojewoda et al. (2015)	RONS accumulation Forcina et al. (2019)
IL-15	Stimulates expression of mitochondrial biogenesis related genes Thornton et al. (2016)	Relieves oxidative stress Li et al. (2014)
MSTN	Promotes mitochondrial fission and biogenesis Ge et al. (2012); Manfredi et al. (2019)	Increases ROS content Sriram et al. (2014); Aravena et al. (2020)
Irisin	Promotes mitochondrial biogenesis Huh et al. (2014)	Induces RONS generation Lee et al. (2015)

However, the mechanism by which mitochondrial dynamics regulate mitochondrial function and redox homeostasis has not been fully revealed. RONS may lead to the S-glutathionylation and S-nitrosylation of the key protein Cys residues in mitochondrial dynamics (Trewin et al., 2018). In addition, RONS stimulates the expression of factors involved in redox regulation and mitochondrial dynamics at the transcriptional level. An example is that mitochondrial ROS levels are controlled by the induction of PGC-1α/β-dependent antioxidant defense mechanisms (Summer et al., 2019), and PGC-1α/β is redox-sensitive and associated with MFN2 regulation (Liesa et al., 2008). AMPK plays a key role in the coupling between mitochondrial dynamics and RONS. AMPK stimulates PGC-1α-dependent mitochondrial biogenesis, which may be important because fusion prevents mitochondrial dysfunction and RONS generation. Conversely, once AMPK is activated, it phosphorylates MFF and DRP1, and mediates mitochondrial fission induced energy deprivation after inhibition of complexes I and III (Trewin et al., 2018). Recent research indicated that the role of AMPK in the downstream of DRP1 mediated mitochondrial fission. DRP1 activity is elevated because of mitochondrial network fragmentation and increased cell survival, which are caused by enhanced activating phosphorylation (Jezek et al., 2018). Of note, the redox regulation of RONS on key enzymes in mitochondrial dynamics can also be mediated by post-translational modifications, such as phosphorylation, ubiquitination and sumoylation (Trewin et al., 2018). Among the core fission/fusion proteins, only DRP1 and possibly OPA1 are post-translationally modified through S-nitrosylation in a redox-sensitive manner (Cho et al., 2009; Bossy et al., 2010). All of these post-translational modifications can be induced by H_2_O_2_, NO^•^, or ONOO^-^, thereby functionally affecting DRP1, OPA1, MFN2, and mitochondrial structure (Willems et al., 2015).

## 6 Mitochondrial Function and Reactive Oxygen/Nitrogen Species Crosstalk Regulate Muscle Stem Cell Fate and Myogenesis

Skeletal muscle stem cells (SMSCs) located between the sarcolemma and basal lamina, also called skeletal muscle satellite cells, which exhibits a remarkable regeneration property after skeletal muscle injury, and are the core of the muscle regeneration process. As aforementioned, there is a complex coupling relationship between mitochondrial function and RONS, and their crosstalk controls the fate decisions of SMSCs and myogenesis. Recently, accumulating evidences suggesting that mitochondrial function, mitochondrial dynamics, and RONS have attracted considerable interest in maintaining and controlling the behavior of SMSCs, including their fate decisions of quiescence, activation, self-renewal, proliferation, migration, alignment, fusion, and differentiation (Rigamonti et al., 2013; Ryall et al., 2015a; Khacho et al., 2016; Le Moal et al., 2017).

### 6.1 Mitochondrial Function Regulates Muscle Stem Cell Fate and Myogenesis

**FIGURE 3:**
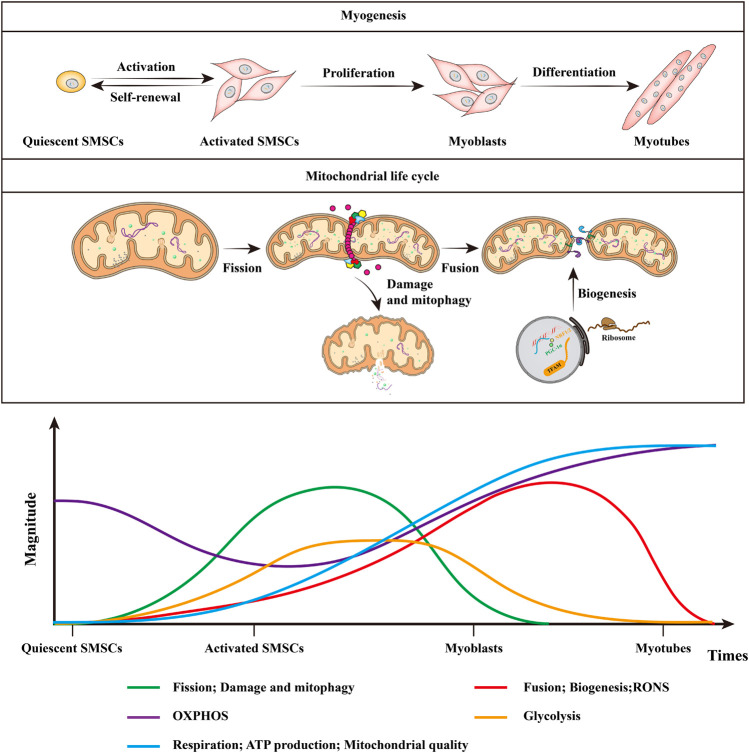
Mitochondrial dynamics and RONS in muscle stem cell fate and myogenesis. Quiescent SMSCs hardly rely on glycolysis, but depend more on mitochondria to generate ATP through β-oxidation of fatty acids and OXPHOS. Mitochondrial metabolism is poorly elucidated in self-renewing SMSCs, whereas myoblasts rely on glycolysis to obtain energy for rapid division. Mitochondrial alteration during myogenesis includes the shift from glycolysis to increased OXPHOS coupled with increased respiration, ATP production, and mitochondrial quality. With myogenic differentiation, there is increased mitochondrial fission, followed by mitophagy, after which the mitochondria are repopulated by mitochondrial biogenesis and fusion. In addition, the quiescent SMSCs have lower RONS levels, RONS levels increase as myoblasts begin to differentiate, and they will decrease in the latter half of the muscle differentiation process. This figure is inspired by Bhattacharya, D. and A. Scime (Bhattacharya and Scimè 2020).

The fate decisions and myogenic differentiation of SMSCs requires extensive intracellular remodeling of mitochondria. SMSCs are characterized by dynamic metabolic reprogramming at different stages of the differentiation process, from predominantly OXPHOS in quiescence to the up-regulation of glycolysis during activation and proliferation, and then to dependence on OXPHOS during terminal differentiation (Bhattacharya and Scimè 2020). The quiescent SMSCs only have only a very few mitochondria tightly packed around the nucleus, their mtDNA and metabolic rate are very low (Latil et al., 2012) (Figure 3). Perhaps because of this characteristic, quiescent SMSCs hardly rely on glycolysis, but depend more on mitochondria to generate ATP through β-oxidation of fatty acids and OXPHOS (Ryall et al., 2015b) (Figure 3). The SMSCs population with low Pax7 levels has more mitochondria and mtDNA and expresses higher levels of myogenic commitment markers. While quiescent SMSCs with reduced mitochondrial density and activity indicate increased stemness markers and express lower levels of myogenic commitment markers (Bhattacharya and Scimè 2020). In neonatal mice, the deletion of skeletal muscle-specific OPA1 results in a significant reduction of quiescent and activated SMSCs, indicating a link between OPA1 and SMSCs self-renewal (Tezze et al., 2017). When activated, SMSCs show a metabolic shift from fatty acid oxidation to a higher glycolysis rate (Ryall et al., 2015b).

Mitochondria in myoblasts are immature, their cristae are underdeveloped, β-oxidation and the overall respiration are maintained at a low level (Sin et al., 2016; Robinson et al., 2019) (Figure 3). Low levels of ETC complexes III, IV and V, mitochondrial proteins, and enzymes maintain the reduced OXPHOS capacity of glycolysis-dependent myoblasts to meet their anabolic demands during proliferation and may also protect them from RONS generated by mitochondrial OXPHOS (Folmes et al., 2012; Hoffmann et al., 2018). The absence of mitochondrial remodeling has been repeatedly proven to reduce the ability of SMSCs myogenic differentiation, and also attenuate the capacity of skeletal muscle tissue regeneration (Wagatsuma et al., 2011; Baechler et al., 2019; Nichenko et al., 2020; Rahman et al., 2020). Under differentiation stimulation, myoblasts must produce ATP at a higher rate to support the intracellular remodeling accompanying differentiation, and the differentiated myoblasts turned into a more oxidative phenotype during this process (Le Moal et al., 2017; Rahman and Quadrilatero 2021). Therefore, the coupling of SMSCs myogenic differentiation and metabolic reprogramming ultimately leads to increased mitochondrial OXPHOS and mitochondrial quality to support the newly formed myotubes. One of the characteristics of early myogenic differentiation is the increase in mitochondrial fission mediated by DRP1 and the subsequent mitophagy mediated by sequestosome 1, they preserve quiescence, maintain stemness and self-renewal, and mediate differentiation (Sin et al., 2016). The activation of autophagy during differentiation serves at least two effects. First, differentiation of stem cells involves extensive cellular remodeling and autophagy to ensure the elimination of unnecessary cellular components. Second, autophagy also helps provide cells with essential building materials, such as amino acids and fatty acids, needed during the cell remodeling by recycling of unnecessary cell components (Lampert and Gustafsson 2020). With reduced mitochondrial elongation, mtDNA content, and mitochondrial biogenesis, the inhibition of DRP1 causes a decrease in myoblasts differentiation (Kim et al., 2013). However, if DRP1 is not inhibited in later stages, myogenic differentiation will not occur (De Palma et al., 2010). With myogenic differentiation, OPA1 mediates mitochondrial fusion and PGC-1α amplifies mitochondrial biogenesis, then forming a dense and elongated mitochondrial network under their synergistic effects, leading to glycolysis and gradually fading (Sin et al., 2016; Bhattacharya and Scimè 2020). This is conducive to the increase of mitochondrial OXPHOS reliance in differentiated myotubes, and is essential for the terminal differentiation of myoblasts (Sin et al., 2016). In addition, ectopic overexpression of PGC-1α in myoblasts can increase mtDNA and the expression of cytochrome c oxidase encoded by mitochondria, thereby enhancing mitochondrial respiration and function (Wu et al., 1999). Compared with myoblasts, differentiated myotubes and myofibers have higher mitochondrial quality and fewer mitochondrial fusion events, which are composed of significant levels of mtDNA, ETC complex proteins and TCA cycle enzymes (Remels et al., 2010; Eisner et al., 2014). Terminally differentiated myofibers need functional mitochondria to maintain the high energy requirements of skeletal muscle.

Compared to myoblasts, the stable contractile structure of mature skeletal muscle, such as the myosin-actin complex, partially limits mitochondrial dynamics by providing a physical barrier (Eisner et al., 2014; Ainbinder et al., 2015). Nonetheless, largescale degradation of these contractile structures can occur during skeletal muscle injury, which may lead to greater remodeling of the mitochondrial network (Eisner et al., 2014; Schiaffino 2017). For example, skeletal muscle-specific DNM1L knockout can lead to reduced mitophagy and severe muscle atrophy (Favaro et al., 2019). However, the deletion of DNM1L in neonatal mice does not cause changes in crucial myogenic regulatory factors, such as MyoD and MyoG (Favaro et al., 2019). In contrast, overexpression of DNM1L and/or FIS1 can cause mitochondrial respiratory dysfunction and diminished mtDNA content as a result of excessive mitophagy (Romanello et al., 2010; Touvier et al., 2015). It should be noted that the potential role of mitochondrial function in symmetric and asymmetric division has not yet been clarified. In short, mitochondrial function and dynamics are the key gatekeepers for muscle stem cell fate decisions and myogenesis.

### 6.2 Reactive Oxygen/Nitrogen Species Regulate Muscle Stem Cell Fate and Myogenesis

Although harmful, RONS produced by electron leakage in ETC can be neutralized by antioxidants and maintained at an optimal level, allowing it to act as a signal molecule (Snezhkina et al., 2019). Although dependent on fatty acid metabolism and OXPHOS, quiescent SMSCs have lower RONS levels, but they express more antioxidants to protect them from the potentially harmful effects of RONS (Aravena et al., 2020; Sriram et al., 2014) (Figure 3). After H_2_O_2_ treatment, quiescent SMSCs have a better survival rate than activated SMSCs, which would cause the accumulation of RONS (Pallafacchina et al., 2010). The down-regulation of PGC-1α in myoblasts increases RONS production, mitochondrial damage, and mitophagy, and leads to poor differentiation (Sin et al., 2016). During myogenic differentiation, RONS induced by mitochondria and NADPH oxidase both are increased (Acharya et al., 2013) (Figure 3). NADPH oxidase is thought to stimulate more mitochondrial RONS via opening mitochondrial ATP-sensitive potassium ions channels, this allows a surge of the potassium ions in the mitochondrial matrix, thereby reducing the mitochondrial membrane potential (Holmuhamedov et al., 1998; Zhang et al., 2001). It should be noted that this effect on membrane potential is limited but not be ignored, although only of 10 mV in the best case (Holmuhamedov et al., 1998). By inhibiting the activity of NADPH oxidase to reduce the production of mitochondrial RONS can prevent its dysfunction (Doughan et al., 2008). In addition, NO^•^ is important for satellite cell activation, self-renewal and myoblast differentiation, which may be closely related to mitochondrial elongation (Buono et al., 2012; Rigamonti et al., 2013). In primary myoblasts, the inhibition of NO synthesis can prevent mitochondrial elongation and myogenic differentiation (De Palma et al., 2010).

Although important, excessive RONS is harmful to myoblasts by targeting mtDNA and mitochondrial function, and cause mitochondrial swelling and disruption (Sestili et al., 2009; Sandiford et al., 2014). Due to the increase in antioxidant enzymes, RONS will decrease in the latter half of the muscle differentiation process, which may be an important characteristic of reducing mitophagy and enabling the repopulation of mitochondria through biogenesis (Le Moal et al., 2017) (Figure 3). In addition, the lack of mitochondrial antioxidant GPx in myoblasts leads to lower cell proliferation and differentiation potential, and primary myoblasts obtained from GPx-deficient mice have poor differentiation ability and impaired myotube formation (Lee et al., 2006). Conversely, the up-regulation of superoxide dismutase in myoblasts promotes the formation of myotubes (Hidalgo et al., 2014). Mechanistically, excessive RONS in myoblasts is considered to elevate nuclear factor kappa B (NF-κB), thereby diminishing the expression level of MyoD and inhibiting myogenic differentiation (Catani et al., 2004; Sandiford et al., 2014). Besides, NF-κB-mediated activation of YY1, a myogenic transcriptional inhibitor, may be another target of the RONS-mediated silencing of myogenic differentiation in myoblasts (Wang et al., 2007). NF-κB can also promote myogenic differentiation through insulin like growth factor II (IGF-2) or p38 MAP kinase, which are both known regulators of myogenic differentiation (Bakkar et al., 2008; Ji et al., 2010). Interestingly, the myoblasts need RONS to exit the cell cycle and initiate the differentiation process via activating p38α MAP kinase (L'honoré et al., 2018). The antioxidant n-acetylcysteine or p38α MAP kinase inhibits RONS, and prevents myogenic differentiation, and elevates the SMSCs pool, indicating the significance of both factors in mediating SMSCs differentiation (Richards et al., 2011; Brien et al., 2013; Bhattacharya and Scimè 2020). Consequently, the positive and negative effects of RONS on SMSCs function may be dose- and time-dependent.

## 7 Concluding Remarks

In this review, we focused on the coupling relationship between RONS and mitochondrial oxidative stress, and systematically described the crosstalk mechanism between skeletal muscle mitochondrial function and RONS and its regulation of muscle stem cell fate and myogenesis. Although it is clear that mitochondria regulate the stem cell fate and function *in vitro* and *in vivo* in most cases, the specific mechanism of mitochondrial function and RONS crosstalk driving muscle stem cell fate and redox homeostasis has not been fully revealed. Therefore, in-depth analysis of these mechanisms would help to reveal how mitochondrial function and RONS coordinately regulate muscle function, which may provide to be valuable information for the development of inhibitory or activating molecules to enhance skeletal muscle regeneration during normal aging or specific diseases.

Moreover, mitochondria can establish direct or indirect connections with other cellular structures, including endoplasmic reticulum, peroxisomes, and lysosomes. At present, mitochondrial biology is now evolving into “organellar biology,” which allows several different organelles to work together to regulate crucial intracellular pathways. However, the function of these interactions in skeletal muscle physiology and pathology is not completely clear. Therefore, further experiments are needed in the future to explore the possible function of “contactology” in skeletal muscle cell signal regulation, and especially to reveal the possible connection with the disease’s formation and development, making it possible to treat humans’ diseases with these organelles as targets.
